# A tale of two islands: evidence for impaired stress response and altered immune functions in an insular pit viper following ecological disturbance

**DOI:** 10.1093/conphys/coaa031

**Published:** 2020-05-03

**Authors:** Mark R Sandfoss, Natalie M Claunch, Nicole I Stacy, Christina M Romagosa, Harvey B Lillywhite

**Affiliations:** 1 Department of Biology, University of Florida, 221 Carr Hall, Gainesville, FL, 32611, USA; 2 School of Natural Resources and Environment, University of Florida, 103 Black Hall, Gainesville, FL, 32611, USA; 3 Aquatic, Amphibian, and Reptile Pathology Program, Department of Comparative, Diagnostic, and Population Medicine, College of Veterinary Medicine, University of Florida, 2015 SW 16th Ave, Gainesville, FL, 32610, USA; 4 Department of Wildlife Ecology and Conservation, University of Florida, 110 Newins-Ziegler Hall, Gainesville, FL, 32611, USA

**Keywords:** Chronic stress, corticosterone, ecological disturbance, Florida cottonmouth, stress physiology

## Abstract

The frequency and intensity of ecological perturbations affecting wild animal populations is expected to increase in the future with animals facing numerous global threats. Seahorse Key is a continental island off mainland Florida that has historically been a major rookery for several species of waterbirds. As a result of an unknown disturbance, the entire rookery abandoned Seahorse Key in April 2015 and shifted nesting activities to nearby Snake Key, resulting in an influx of food resources in the form of fish carrion to resident Florida cottonmouth snakes (*Agkistrodon conanti*), while snakes on Seahorse Key experienced a drastic reduction in food resources. Our objective was to assess plasma corticosterone concentrations, corticosterone negative feedback using dexamethasone, blood glucose, body condition, packed cell volume, natural antibody agglutination, white blood cell counts and ratios and erythrocyte sedimentation rate to characterize the long-term effects of differential resource availability in these two snake populations 3 years after this major ecological disturbance. We collected blood samples at three time points from cottonmouths on Seahorse Key (*n* = 6 individuals) and Snake Key (*n* = 13 individuals) in fall 2018. In due consideration of the small sample size, our study shows evidence that 3 years after the shift in waterbird nesting Seahorse Key cottonmouths exhibit a dampened acute stress response and presumptive impaired innate immune functions relative to cottonmouths on Snake Key. These results highlight the context-dependent nature of biomarkers and implicate the significant decrease in food resources on Seahorse Key in altering hormonal stress responses and innate immune functions, possibly leading to unknown long-term downstream effects. This study assessed the response of a wild population of pit viper to ecological disturbance *in situ* with the aim to improve our understanding of how animals cope with such perturbations and improve our capacity to make informed decisions for conservation.

## Teaser

There is an increasing need for conservation tools that allow scientists and managers to assess the physiological and immune health of free-ranging animal populations. We evaluated several commonly used biomarkers to characterize the health of two insular snake populations with markedly different availability of food resources after ecological disturbance.

## Introduction

‘It was the best of times, it was the worst of times’ is the opening line of the famous novel *A Tale of Two Cities* (Dickens 1859) and is thought to describe the paradoxical nature of a world where some are thriving while others are toiling. This paradox applies to the broader global environment as animals are being faced with increasing ecological disturbance, e.g. habitat destruction, biological invasion and changes in resource availability (Wingfield 2008; Wingfield *et al*. 2011; Rocha *et al*. 2018; IPBES 2019). The ability to respond appropriately to stress caused by ecological disturbance determines individual survival (Romero and Wikelski 2001, 2010) and consequently whether a population persists through time. The need to maintain a balanced energy budget while responding to a stressor can cause animals to divert energy away from other essential physiological processes, such as immune functions (French *et al*. 2007; Dhabhar 2009; Martin 2009), growth (Laugero and Moberg 2000), and reproduction (Greenberg and Wingfield 1987). Investigating animal populations following environmental disturbance may provide important insights into the ability of animal populations to respond to global stressors and threats (Wingfield 2008; Somero 2010; Wingfield *et al*. 2011).

To date, several physiological biomarkers of energy status, hormonal regulation and immunity have been used to assess population health (Wikelski and Cooke 2006; Deem *et al*. 2008; Busch and Hayward 2009; Dantzer *et al*. 2014). However, the value of these physiological biomarkers as metrics for characterizing the response of a population to disturbance remains an open question (Fefferman and Romero 2013; Gangloff *et al*. 2017). The primary physiological biomarker used by researchers and wildlife managers to assess stress on populations is the concentration of glucocorticoid hormones (primarily corticosterone or cortisol; Jessop *et al*. 2013). The release of corticosterone occurs via the hypothalamic–pituitary–adrenal axis (HPA) in the majority of vertebrates, including reptiles (Sapolsky *et al*. 1986). Increased blood concentrations of corticosterone above some baseline level are assumed to be indicative of a stress response. Short-term or acute increases in corticosterone may be an adaptive response to perturbations in homeostasis (i.e. allostasis; see Wingfield *et al*. 1998; Sapolsky *et al*. 2000), which can trigger an emergency life-history stage that mediates energetic trade-offs among physiological mechanisms by suppressing processes not immediate to survival, thus allowing an individual to endure a stressor (Romero and Wikelski 2001; Wingfield 2003; Schoech *et al*. 2013). However, when animals are exposed to long-term or chronic stressors, this can lead to the disruption of proper HPA function, and the ability of the HPA axis to end the stress response via negative feedback has been used more recently to measure chronic stress and may be an informative assessment of the stress response (Romero 2004; Romero and Wikelski 2010; Zimmer *et al*. 2019; Lattin and Kelly 2020). Much work has been done to estimate the correlation between both baseline and stress-induced concentrations of corticosterone and organismal fitness, but this relationship is not well-defined (Breuner *et al*. 2008; Bonier *et al*. 2009; Crespi *et al*. 2013). The acute corticosterone response may better predict an organism’s ability to cope with stressors than do baseline levels (Kitaysky *et al*. 2001; Breuner *et al*. 2008; Dickens and Romero 2013; McCormick *et al*. 2015; Neuman-Lee *et al*. 2015; Taff and Vitousek 2016). In summary, corticosterone concentrations in various settings appear to be context-dependent and are potentially informative, but principally when combined with concurrent additional measures of physiological health (Breuner *et al*. 2013).

Other physiological biomarkers that have been used to assess health in free-ranging animals include body condition index, blood glucose and packed cell volume. Body condition index has been routinely used as a proxy for individual feeding history and food availability (Jayne and Bennett 1990; Bonnet and Naulleau 1995). Blood glucose is an indicator of energy availability and is thought to increase following a stressor via initiation of glycogenolysis and gluconeogenesis, mediated in part by corticosterone (Fujiwara *et al*. 1996; Sapolsky *et al*. 2000; Neuman-Lee *et al.*, 2020). Packed cell volume is the proportion of blood volume occupied by packed red blood cells relative to plasma and is commonly used in vertebrates as a comparative index of general health (Hoi-Leitner *et al*. 2001). Although decreased packed cell volume (i.e. anaemia) can be indicative of chronic poor nutrition (Dunlap 1995), it is non-specific and can also result from numerous other conditions (Stacy *et al*. 2011).

**Figure 1:**
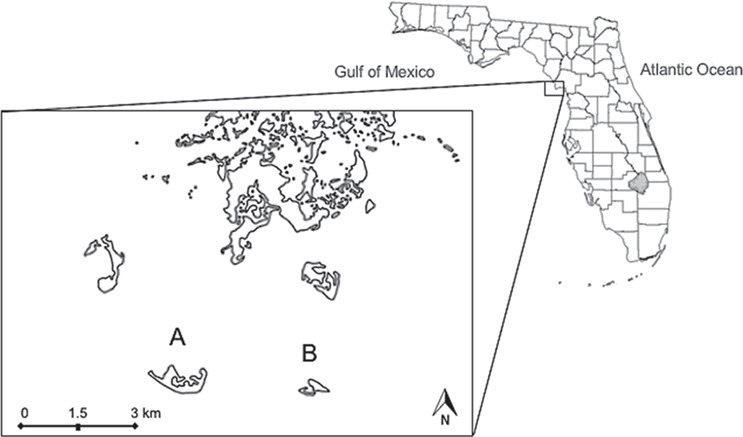
Aerial view of the study area including Seahorse (**A**) and Snake Key (**B**) located in the Gulf of Mexico off the coast of mainland Florida. County boundaries delineated on map of Florida

Additionally, analyses of white blood cell counts and ratios, erythrocyte sedimentation rate, natural antibody (NAb) activity and presence of haemoparasites by blood film evaluation can be used to assess general immune function and overall health. Evaluation of peripheral white blood cell counts can be informative in the diagnostic assessment of chronic stress in wild animal species (Davis *et al*. 2008; Davis and Maney 2018). White blood cell counts and heterophil to lymphocyte (H: L) ratio also provide a gross correlative measure of immune functions and current inflammatory status. Erythrocyte sedimentation rate is a non-specific test of immune function that indicates an active inflammatory response (Kumar *et al*. 2010; Rosenberg *et al*. 2018). Sedimentation rate increases during acute inflammation as inflammatory proteins cause red blood cells to aggregate together and fall faster (Kumar *et al*. 2010). Quantification of NAb agglutination activity in the blood plasma is indicative of the ability of animals to effectively respond to a novel antigen (Matson *et al*. 2005). NAbs form a functional link between the innate and acquired parts of the humoral immune system acting as a first line of defence against pathogens (Ochsenbein and Zinkernagel 2000) and have been linked to increased survival in fish and poultry (Kachamakova *et al*. 2006; Star *et al*. 2007). Although the adaptive function of NAbs are not well understood in reptiles, they are thought to be a key immune defence as NAbs are maintained or increased with age (Palacios *et al*. 2011; Ujvari and Madsen 2011; Zimmerman *et al*. 2017), may vary with environmental exposure (Palacios *et al*. 2011) and can offset investment in acquired antibody production after antigen exposure (Sandmeier *et al*. 2012). Parasite burden can be used as an indicator of individual health in wild animals (Moller *et al*. 1999). Both endo- and ectoparasites are frequently prevalent in reptiles (Jacobson 2007), and individuals with a high infestation of parasites are thought to be in general poor health. However, the relationship between parasite burden, immune function and stress is not clearly understood (Sanchez *et al*. 2018).

The physiological and immune responses of organisms appear to be relatively context-dependent, and there is no one single biomarker that predicts previous or ongoing exposure to chronic stress (Dickens and Romero 2013). Investigations into the ability of biomarkers to characterize the response of populations to environmental change are particularly informative for researchers and wildlife managers interested in making decisions concerning conservation (Dantzer *et al*. 2014). Experimental studies testing the response of physiological biomarkers such as corticosterone to natural disturbance on free-ranging organisms are relatively rare owing to both practical and ethical implications of inducing long-term stress on wild animal populations *in situ* (Sopinka *et al*. 2015; Claunch *et al*. 2017a). In addition, studies of immune functions in free-ranging organisms under environmentally induced stress has presented a complex picture of animal physiology and tradeoffs (e.g. Saino *et al*. 1997; Sorci *et al*. 1997; Palacios *et al*. 2013) and requires further study. Laboratory evidence suggests that immune function is directly associated with body condition and dietary preferences, both of which can change under stress (Chandra and Newberne 1977; Klasing 1988; Martí *et al*. 1994; Foster *et al*. 2006).

Florida cottonmouth snakes, *Agkistrodon conanti* (Viperidae) Gloyd 1969, have a unique trophic association with colonially nesting waterbirds of several species within Pelecaniformes on two islands off the western coast of peninsular Florida in the Gulf of Mexico (Fig. 1) (Lillywhite and McCleary 2008). These two islands, Seahorse Key and Snake Key, are a part of the Cedar Keys National Wildlife Refuge that was established in the 1920s to provide nesting habitat for the tens of thousands of waterbirds that nest seasonally (March to November) on the islands. The majority (95%) of nesting has occurred on Seahorse Key since 1964 (US Fish and Wildlife Service, unpublished data). The waterbird rookery provides substantial food resources to cottonmouth snakes in the form of fish carrion scavenged from the forest floor after being accidentally dropped or regurgitated by birds (Wharton 1969; Lillywhite and McCleary 2008). The input of allochthonous marine resources has led to a relatively high abundance of cottonmouths on Seahorse Key (5–55 snakes/ha, mean ~10 snakes/ha, Wharton 1969; Lillywhite and Sheehy, 2019).

**Table 1:** Summary table of predictions for the relative trends of physiological biomarkers for Florida cottonmouth snakes (*Agkistrodon conanti*) from Seahorse and Snake Key

Physiological biomarker	Seahorse Key	Snake Key	Reference
Baseline CORT	High	Low	Sapolsky *et al*. 2000
Acute CORT	High	High	Sapolsky *et al*. 2000; Dickens and Romero 2013
DEX CORT	High	Low	Romero 2004; Romero and Wikelski, 2010; Nevarez *et al*. 2011
Glucose	Low	High	Fujiwara *et al*. 1996; Gangloff *et al*. 2017
Body condition index	Low	High	Jayne and Bennett 1990; Bonnet and Naulleau 1995
Packed cell volume	Low	High	Dunlap 1995; Hoi-Leitner *et al*. 2001
NAb	Low	High	Sparkman and Palacios 2009; Palacios *et al*. 2011; Holden *et al*. 2019
ESR	High	Low	Kumar *et al*. 2010; Rosenberg *et al*. 2018
WBC count	Low	High	Davis *et al*. 2008; Davis and Maney 2018
H:L (HA:L)	Low	Low	Davis and Maney 2018

The associated literature reference for justification of prediction is provided for each physiological biomarker in the ‘reference’ column. Seahorse Key has low food abundance and Snake Key has high food abundance. (CORT = corticosterone, DEX = dexamethasone, NAb = natural antibody activity, ESR = erythrocyte sedimentation rate, WBC = white blood cell, H:L heterophil to lymphocyte ratio, HA:L = heterophil plus azurophil to lymphocyte ratio)

Unexpectedly, in April of 2015, the entire colony of nesting waterbirds on Seahorse Key abandoned their nests for unknown reasons and have not returned. A third or more of these birds, estimated 3000–5000 individuals, shifted nesting to Snake Key in 2015 and have continued nesting on that island (U.S. Fish and Wildlife Service, unpublished data). Snake Key is located ~ 2.5 km to the east of Seahorse Key (Fig. 1) and the two islands are separated by seawater, which is a significant barrier to the movement of cottonmouths between the islands (Sandfoss and Lillywhite 2019). Consequently, cottonmouths on Snake Key have recently received a large influx of fish carrion, while snakes on Seahorse Key have experienced a drastic reduction of food resources following bird-abandonment (Sandfoss *et al*. 2018). The loss of available fish carrion on Seahorse Key is correlated with declines in snake body condition (Sandfoss *et al*. 2018) and abundance (Lillywhite and Brischoux 2012; Sandfoss *et al*. 2018). The declines in abundance are likely due to increased mortality as documented by increased numbers of carcasses and 50% mortality of radio-tracked snakes on Seahorse Key in 2018/2019 (Sandfoss *et al*. 2018; M.R. Sandfoss unpubl. data). Additionally, behavioural changes in the Seahorse Key cottonmouths were observed as snakes moved away from abandoned rookery trees (M.R. Sandfoss unpubl. data), and the first instances of cannibalism were documented in this population (never observed during decades of previous research; see Carr Jr 1936; Wharton 1969; Sheehy III *et al*. 2017; Lillywhite and Sheehy III 2019). Corresponding physiological effects of abandonment had not been documented.

The sudden loss of bird-provided food resources on Seahorse Key provides a unique opportunity to measure physiological biomarkers for the characterization of the long-term response of cottonmouths to substantially changing food resources 3 years after a major ecological perturbation. A comparative approach of biomarkers between cottonmouths from Seahorse and Snake Key is novel in an *in situ* setting and is an ideal opportunity to elucidate the responses of snakes to ecological disturbance and identify physiological metrics for health responsive to disturbance in free-ranging populations. Predictions for the response of each physiological biomarker to the shift in food resources for Seahorse and Snake Keys are provided in Table 1. Our objective was to assess plasma corticosterone, blood glucose, packed cell volume, NAb agglutination, white blood cell counts and ratios and erythrocyte sedimentation rate to characterize the long-term effects of differential resource availability in these two snake populations 3 years after ecological disturbance. The snake populations investigated in this study are part of a well-studied system (Carr, 1936; Wharton 1969; Lillywhite and Sheehy, 2019).

## Methods

### Animals and study site

Seahorse and Snake Key are 67- and 15-ha continental islands, respectively, that lie ~6 km from mainland Florida (Fig. 1). Both islands have similar habitat, consisting primarily of mixed upland hardwood hammock and extensive mangrove stands, surrounded by brackish waters containing sea grass, sand flats and oyster beds.

We sampled insular cottonmouths at the end of the waterbird nesting season between September 5 and October 12 of 2018 from Seahorse and Snake Key. Because of the impacts of the shift in nesting by colonial waterbirds, the abundance of snakes available for sampling differed on the two islands, and we were unable to sample equal numbers of snakes on each island. Due to the significant decline in the snake population on Seahorse Key (Sandfoss *et al*. 2018), and despite increased searching effort, it is increasingly difficult to find live, healthy cottonmouths on the island. Moreover, we did not want to disturb the entirety of the surviving population on Seahorse Key. Our sampling was also hindered by a Category 5 hurricane (‘Michael’) that passed through our study area on 9 October 2018.

A total of 19 adult Florida cottonmouth snakes were captured from Seahorse (female *n* = 2, male *n* = 4, mass 323–1432 g) and Snake Key (female *n* = 6, male *n* = 7, mass 440–2178 g). Of the 19 individuals that were captured, 11 were males and 8 were non-gravid females. Cottonmouths were opportunistically captured on Seahorse and Snake Key during searches of the inner hammock and beaches between sunset and 21:00 EST. Snakes were placed in buckets, and body mass was determined using a spring scale. Snakes were then immediately secured in a plastic restraining tube for blood sampling (see below) and body temperature measured via a thermocouple temperature probe (Traceable Products™, Webster, TX, USA; #14-649-81) inserted ~ 1 cm into the cloaca (baseline Snake Key mean (± SD) = 29.8 ± 0.9°C; baseline Seahorse Key mean (± SD) = 28.5 ± 1.8°C).

After completion of blood sampling at three time points in the field as described below, snakes were transported to the laboratory at the University of Florida, ~90 km from the study site where we measured snout-vent length (SVL) and identified sex. To prevent resampling individuals, all snakes were maintained in the laboratory and released at the original sites of capture after the study’s completion.

All work was completed under the approval of the University of Florida’s Institute of Animal Care and Use Committee (study #201809079). Animals were captured, handled and housed according to Florida venomous reptile license #411-133 587 and U.S. Fish and Wildlife Service federal special use permit #41511-14-10.

### Body condition index

Body condition index was calculated using residuals from a linear regression of log10-SVL and log10-body mass of all sampled snakes (Jayne and Bennett 1990).

### Endpoints of blood analysis

#### Corticosterone assay

Immediately upon capture, while snakes were restrained in a plastic tube a baseline blood sample of < 1 mL was aseptically collected from the caudal vein of cottonmouths with a heparinized 25-gauge needle. Baseline samples were collected within a mean (± SD) time of 4.1 ± 2.0 min at first contact with the animal (Romero and Reed 2005); a critical inflection point has not been clearly identified in reptiles (Manzo *et al*. 1994; Romero and Reed 2005; Tylan *et al*. 2020) and is likely species-specific.

Blood was then transferred to a heparinized 400-μL microcentrifuge tube (BD, Franklin Lakes, NJ, USA; #365965) and kept insulated on ice until further processing. Blood samples were processed upon return to the laboratory, usually within 3 h of initial capture of all snakes. Plasma was collected after centrifugation of whole blood at 10000 rpm for 2 min (Fisher Scientific, Hampton, NH, USA; Micro-Centrifuge Model 59A) and stored at −80°C until further processing for analyses.

We obtained all baseline blood samples between 1913 and 2100 h (Snake Key mean (± SD) = 2003 ± 28; Seahorse Key mean (± SD) = 1958 ± 34) to minimize diel variation in corticosterone (Romero and Wikelski 2006). After baseline sampling, snakes were exposed to an ‘acute stressor’ which consisted of a standardized acute restraint protocol (Claunch *et al*. 2017a). The restraint protocol consisted of holding snakes individually inside buckets for 78 ± 16 min at ambient conditions. At the end of the period of restraint, blood and temperature (acute Snake Key mean (± SD) = 28.9 ± 1.1°C; acute Seahorse Key mean (± SD) = 29.2 ± 1.0°C) were collected as described above to evaluate the ability of insular cottonmouths to respond to an acute stressor (defined as ‘acute stress’ sample).

Immediately after the acute stress sample was collected, snakes received an intraperitoneal injection of dexamethasone (VetOne®, Boise, ID, USA; #501012) at 1 μg/g body mass (Romero and Wikelski 2010). A third and final blood sample was collected 60 min after the injection of dexamethasone (120 min post-capture, ‘DEX’ sample). Dexamethasone binds to glucocorticoid receptors and should ultimately cause a decrease in the body’s synthesis of glucocorticoids if the HPA axis is functioning properly (Sapolsky *et al*. 1986; Romero 2004). Temperature was also recorded at the DEX sampling time point as described above (DEX Snake Key mean (± SD) = 28.0 ± 1.7°C; DEX Seahorse Key mean (± SD) = 28.5 ± 2.1°C).

Circulating plasma concentration of corticosterone (in ng/mL) at each sampling point (baseline, acute stress, DEX) was measured using an enzyme immunoassay kit (Arbor Assay, Ann Arbor, MI, USA; #KO14-H5). The kit was validated for use with *A. conanti* via serially diluting pooled *A. conanti* plasma and assessing parallelism and quantitative recovery. Average recovery was 93%. Plasma samples were diluted 1:100 with assay buffer and run in duplicate according to protocol provided by the kit manufacturer. The optical density of each well was read at 450 nm (BioTek, Winooski, VT, USA; Model Epoch). The intra-assay coefficient of variation (2.58%) was calculated from the variation in duplicate plasma samples from each individual, averaged across each plate. The inter-assay coefficient of variation (6.4%) was calculated from the standard curves on each plate.

#### Glucose

We assessed whole blood glucose of snakes and the real-time effect of changes in corticosterone on glucose mobilization. Glucose was measured (in mg/mL) immediately after collection of all blood samples with a commercially available glucometer (FreeStyle Lite Glucometer, Abbot Diabetes Care, Alameda, CA, USA) (Breuner *et al*. 2013; Gangloff *et al*. 2017). The majority of glucose values for cottonmouths at the baseline sample was below the manufacturer-indicated lower limit of detection (20 mg/dL; *n* = 17 out of 19 baseline samples) which precluded analyses of glucose at baseline.

#### Packed cell volume

Packed cell volume was determined after spinning ~50 μL of whole blood in a 70 μL heparinized capillary tube for 5 min at 10 000 rpm on a centrifuge (Thermo Fisher Scientific, Waltham, MA, USA; IEC MB Centrifuge) with a standardized capillary tube reader card (Lancer, St. Louis, MO, USA).

#### Haemolysis–haemoagglutination assay

To assess NAb agglutination and lysis in plasma, we modified a previously described protocol (Matson *et al*. 2005). Modifications to the Matson protocol included adding 20 μL of 2% sheep blood (Hemostat Laboratories, Dixon, CA; #SBH100) suspension to serially diluted plasma samples (1:1 to 1:1024, 20 μL in each well) plated in duplicate. Haemolysin was added to half of the final column as a positive control. Plates were incubated for 90 min at 22°C, which is within the active Tb range of cottonmouths during the sampling period (H.B.L. unpublished data). The incubation periods for agglutination and lysis and scoring procedures were as in Matson *et al*. (2005), except plates were scanned at 600 DPI (Hewlett-Packard, Palo Alto, CA, USA; OfficeJet 4650). Some samples were run singly due to insufficient plasma (*n* = 15 samples).

#### Erythrocyte sedimentation rate

Sedimentation rate increases during acute inflammation as inflammatory proteins cause red blood cells to aggregate together and fall faster (Kumar *et al*. 2010). Approximately 50 μL of whole blood was drawn into a 70-μL heparinized capillary tube and oriented vertically for 60 min. The erythrocyte sedimentation rate was scored as the length of tube (in mm) of blood volume that was no longer occupied by red blood cells.

#### White blood cell concentrations and ratios

Upon arrival to the laboratory, within 3 h of blood draw, we prepared three blood films from well-mixed whole blood from each sample. Slides were stained with Wright-Giemsa (Harleco®, EMD Millipore, Billerica, MA, USA) and evaluated in a consistent manner by one clinical pathologist (N.S.) who was blinded to animal IDs. A white blood cell estimate (K/μL) was performed using a semi-quantitative assessment (Weiss 1984), in addition to a 200 white blood cell differential (heterophils, basophils, eosinophils, azurophils, lymphocytes and monocytes), and morphologic evaluation of white blood cells, red blood cells and thrombocytes. Heterophil to lymphocyte (H:L) and heterophil+azurophil (A) to lymphocyte ratios (HA:L) were assessed. To quantify evidence of erythroid regeneration, immature red blood cells per 100 mature red blood cells were recorded. Furthermore, whole blood collected at each time point (baseline, acute, DEX) from all snakes was used to prepare blood films which were scanned for the presence of haemoparasites.

### Statistical analyses

During statistical analyses, data were tested for assumptions required by parametric tests and were either transformed to meet assumptions of normality and equal variance or non-parametric tests were used. All of our measures were each analyzed separately for fixed effects of population (Seahorse and Snake Key), sampling point (baseline, acute stress, DEX) and their interaction using a linear mixed-effects model fit by maximum likelihood with the nlme R package (Pinheiro *et al*. 2018). The individual identifier of snakes was included as a random effect in all models. Residuals of models were tested for normality using Shapiro’s test and plots of residuals inspected for heteroscedasticity. We did our best to control for the possible effects of covariates in our study design (e.g. sampling time, body temperature) and thus have not included covariates in our models. This was necessary to avoid overparameterization because of our sample sizes.

We quantified the negative feedback ability of animals via measures of corticosterone across sampling time points. There are multiple approaches to the calculation of the change in corticosterone concentrations to measure negative feedback success (Lattin and Kelly 2020). Here we report negative feedback success calculated by five different methods: (i) raw corticosterone post dexamethasone injection, (ii) difference from baseline, (iii) relative difference from baseline, (iv) reduction from acute stress sample and (v) relative reduction from acute stress sample and then compared these measures between Seahorse and Snake Key using the non-parametric Wilcoxon rank-sum test. We then also performed a multivariate Spearman’s correlation test between the five negative feedback measures.

Packed cell volume was only collected at the baseline sampling point and was compared for differences between populations and sex using an analysis of variance (ANOVA). Body condition index values of snakes were compared for differences between population and sex using Student’s *T* tests.

To determine if individual metrics of immunity assays were correlated with each other (Sparkman *et al*. 2014; Neuman-Lee *et al.*, 2019), we combined all individuals from both populations and performed multivariate Spearman’s correlations of log10-corticosterone, log10-glucose, body condition index, log10-NAb, square-root-erythrocyte sedimentation rate, log10-total white blood cell count, log10-H:L ratio, log10-HA:L ratio and log10-immature red blood cells for each sampling point (baseline, acute stress, DEX). Glucose was not included in Spearman’s correlation tests of baseline samples. A few individuals were missing data (NAb *n* = 2, erythrocyte sedimentation rate *n* = 4) due to insufficient volume of blood collected at one or more sampling points. Therefore, after confirming the missing values were missing completely at random, nulls were replaced with imputed values using predicted mean matching (Little 1988) calculated using the ‘mice’ package in program R (van Buuren and Groothuis-Oudshoorn 2011), only for the correlation tests.

Descriptive statistics are reported as means (±SD) and all statistical tests were performed in Program R (version 3.4.3) with alpha set to 0.05.

## Results

### Body condition index

The body condition index data of cottonmouths from Snake Key (- = 0.03 ± 0.06) were relatively higher compared to snakes from Seahorse Key (- = −0.07 ± 0.06) (*t* = −3.395, d.f. = 9.4, *P* = 0.007). There was no detectable difference between sexes (male: - = −0.026 ± 0.06, female: - = 0.04 ± 0.09) (*t* = 1.731, d.f. = 11.8, *P* = 0.110). Body condition was moderately and positively correlated with NAb (*ρ* = 0.51) and corticosterone (*ρ* = 0.55) at the DEX sampling point only (Fig. 3).

### Endpoints of blood analysis

#### Corticosterone

**Figure 2:**
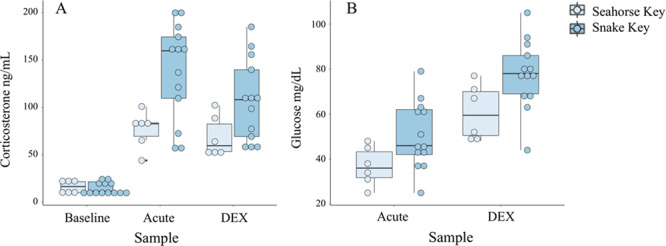
Box plots of (**A**) plasma corticosterone (in ng/mL) and (**B**) blood glucose (in mg/dL) collected at three sampling time points (baseline, acute and following injection of dexamethasone (DEX)) from Florida cottonmouth snakes (*Agkistrodon conanti*) on Seahorse (*n* = 6) and Snake Keys (*n* = 13). Seahorse Key has low food abundance, and Snake Key has high food abundance. Individual dots represent values for each individual snake

**Figure 3:**
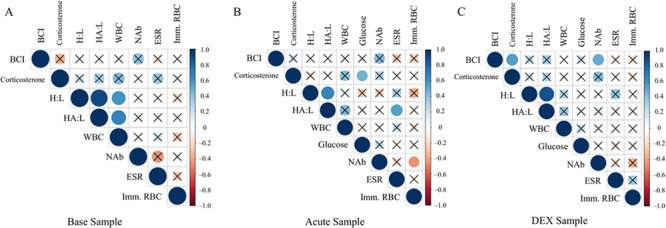
(panels **A**–**C**) Spearman’s correlation tests of immune and physiological measures from the pooled data of Florida cottonmouth snakes (*Agkistrodon conanti*) from Seahorse and Snake Key combined (*n* = 19). Seahorse Key has low food abundance and Snake Key has high food abundance. The size of each circle represents the level of correlation between two variables with those circles marked with an ‘X’ being non-significant tests at an alpha level of 0.05. Warm to cool colour scale bar describes the rho correlation coefficient (BCI = body condition index, H:L = heterophil to lymphocyte ratio, HA:L = heterophil plus azurophil to lymphocyte ratio, WBC = total white blood cell count, NAb = natural antibodies, ESR = erythrocyte sedimentation rate, Imm. RBC = immature red blood cells)

**Figure 4:**
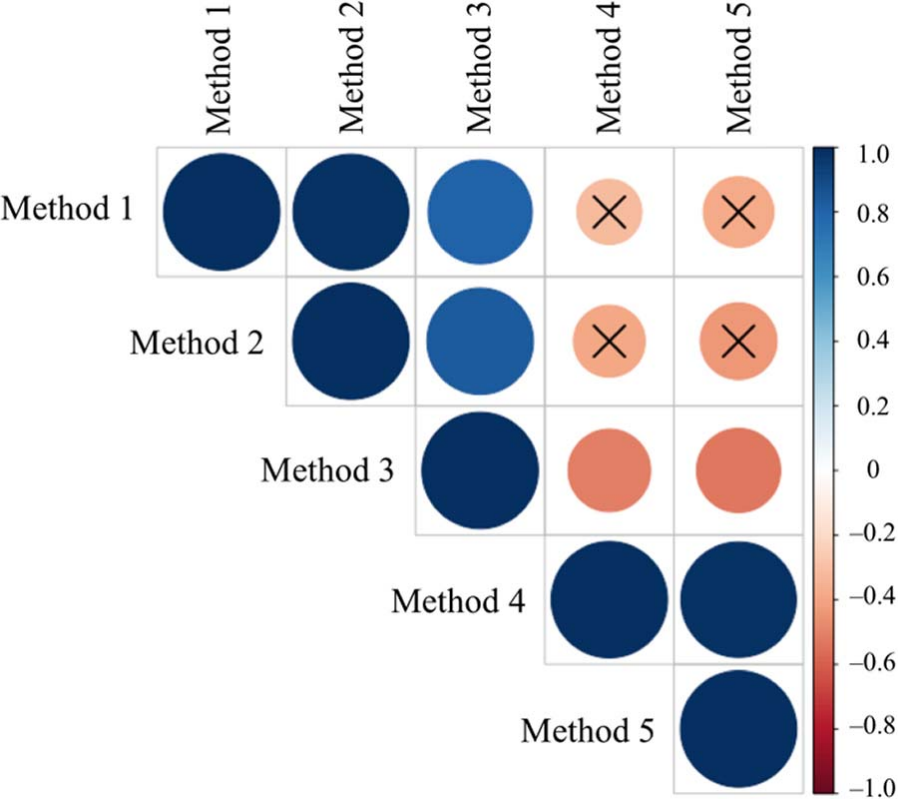
Spearman’s correlation tests of five methods for calculating negative feedback success following injection with dexamethasone in Florida cottonmouth snakes (*Agkistrodon conanti*) from Seahorse and Snake Keys combined (*n* = 19). Seahorse Key has low food abundance and Snake Key has high food abundance. The size of each circle represents the level of correlation between two variables with those circles marked with an ‘X’ being non-significant tests at an alpha level of 0.05. Warm to cool colour scale bar describes the rho correlation coefficient. Please see Methods section for a full description of each of the five calculation methods

Corticosterone concentrations (in ng/mL) increased from baseline sample (Seahorse Key - = 15.97 ± 6.6 ng/mL, Snake Key - = 14.37 ± 6.5 ng/mL) to the acute stress sample (Seahorse Key - = 76.58 ± 19.6 ng/mL, Snake Key - = 138.27 ± 51.1 ng/mL), with cottonmouths from Snake Key producing a much larger magnitude increase in plasma corticosterone, and plateauing at the DEX sample (Seahorse Key - = 68.79 ± 21.6 ng/mL, Snake Key - = 107.08 ± 43.3 ng/mL) (Table 2, Fig. 2a). The percent change between acute stress sample values of corticosterone and values following DEX injection did not significantly differ (Wilcoxon rank sum test *W* = 35, *P* = 0.766) between snakes on Seahorse (- = 8.3 ± 22.6%) and Snake Keys (- = 12.1 ± 43.5%). Corticosterone was moderately positively correlated with glucose at the acute sampling point (*ρ* = 0.47) and, as mentioned above, correlated positively with body condition (*ρ* = 0.55) (Fig. 3). Comparisons of negative feedback success using each of the five calculation methods found a significant difference between the two populations using Methods 1 and 2 only (Table 4). Spearman’s correlation showed only Methods 4 and 5 did not correlate with Methods 1 and 2 (Fig. 4).

**Table 2:** Summary table of mixed effects model for each measurement of health from all sampled Florida cottonmouths snakes (*Agkistrodon conanti*). Each coefficient is calculated in relation to Seahorse Key Population, Baseline Sample, or Snake Key * Baseline Sample. Population refers to differences between population (Seahorse and Snake Keys). Seahorse Key has low food abundance and Snake Key has high food abundance. Sample refers to the three sampling time points (baseline, acute, and 60 minutes after the injection of dexamethasone (120-minutes post-capture, ``DEX'' sample). An asterisk indicates an interaction term. (NAb = Natural antibodies, ESR = Erythrocyte sedimentation rate, WBC = White blood cells, H:L = Heterophil to lymphocyte ratio, HA:L = Heterophil +Azurophil to lymphocyte ratio, RBC = Red blood cells)

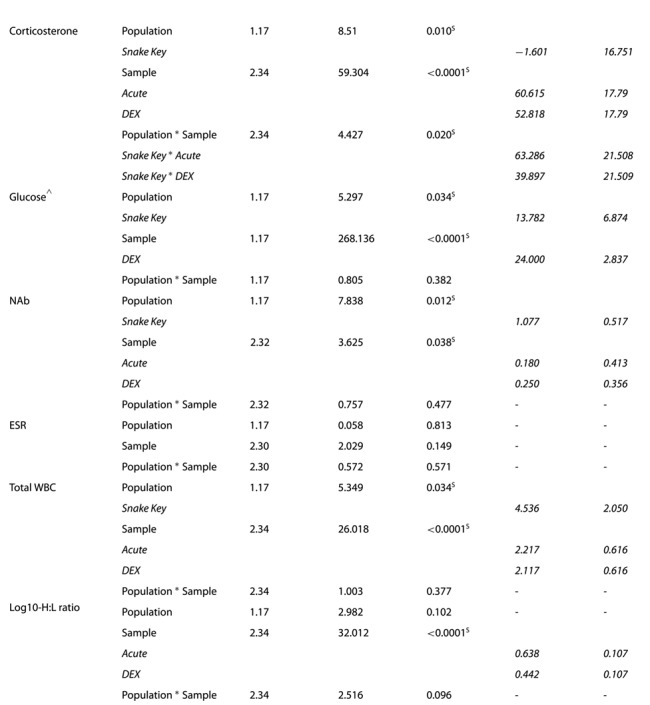
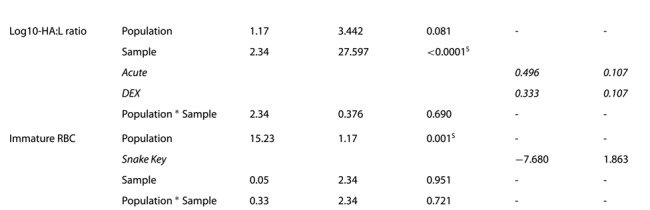

^S^ indicates variables that were found to be statistically significant at the 0.05 level.

^^^Glucose was only measured at two sampling time points (Acute and DEX).

#### Glucose

Glucose increased significantly with each successive sampling point (Table 3, Fig. 2b), although we cannot enumerate baseline means beyond being <20 mg/dL. Snake Key experienced a greater increase in glucose with each successive sampling than Seahorse Key (Table 3, Fig. 2b). Additionally, as noted above glucose was positively correlated with corticosterone (*ρ* = 0.47) at the acute sampling point (Fig. 3).

#### Packed cell volume

The results of the two-way ANOVA for packed cell volume found no significant effects of population (*F*_1,16_ = 3.850, *P* = 0.067) or sex (*F*_1,16_ = 0.006, *P* = 0.938).

#### Haemolysis–haemoagglutination assay

**Figure 5:**
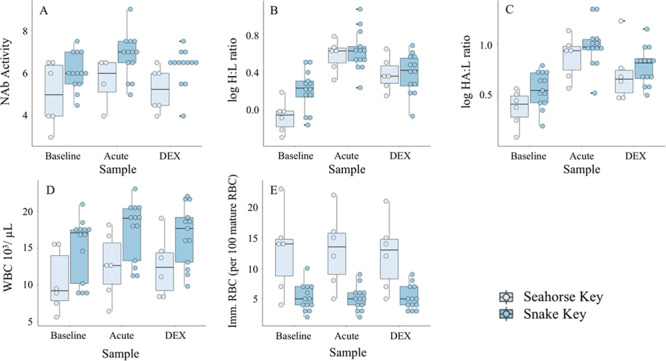
Box plots of (**A**) natural antibody (NAb) agglutination activity, (**B**) log10-heterophil to lymphocyte ratio (H:L), (**C**) log10-heterophil plus azurophil to lymphocyte ratio (HA:L), (**D**) total white blood cell counts (WBC) and (**E**) number of immature red blood cells (Imm RBCs) per 100 mature red blood cells calculated from whole blood samples collected at three sampling time points (baseline, acute and 60 min after injection of dexamethasone (DEX)) from Florida cottonmouth snakes (*Agkistrodon conanti*) on Seahorse (*n* = 6) and Snake Keys (*n* = 13). Seahorse Key has low food abundance, and Snake Key has high food abundance. Individual dots represent values for each individual snake

Lytic activity of the complement in *A. conanti* was not detectable with this assay (see Matson *et al*. 2005; Merlo *et al*. 2016), therefore only agglutination activity (NAb) results are reported and analyzed. Agglutination arises from NAbs only and lysis reflects an interaction between NAbs and complement enzymes (Matson *et al*. 2005). Cottonmouths from Snake Key had better agglutination ability (- = 6.31 ± 1.2) than snakes from Seahorse Key (- = 5.01 ± 1.3) (Table 2, Fig. 5a). Agglutination ability increased from baseline (- = 5.62 ± 1.3) to acute stress samples (- = 6.17 ± 1.2) and decreased again at the DEX sample (- = 5.89 ± 1.2) but remained elevated relative to baseline ability (Table 3, Fig. 5a). NAb activity was positively correlated with body condition (*ρ* = 0.51) at the DEX sampling point and weakly negatively correlated with immature red blood cells (*ρ* = −0.46) at the acute sampling point (Fig. 3).

#### Erythrocyte sedimentation rate

There were no significant differences found in erythrocyte sedimentation rate between populations or across sampling points or their interaction (Tables 2 and 3). Erythrocyte sedimentation rate was moderately positively correlated with HAL ratio (*ρ* = 0.55) at the acute sampling point (Fig. 3).

#### White blood cell counts and ratios

Total white blood cell count increased at acute stress sample and plateaued at DEX sampling points (baseline - = 12.59 ± 1.5 K/μL, acute stress - = 15.14 ± 1.3 K/μL, DEX - = 14.79 ± 1.4 K/μL) (Table 2, Fig. 5d). Total white blood cell count was significantly lower in cottonmouths from Seahorse Key (- = 11.17 ± 1.4 K/μL) compared to Snake Key (- = 15.81 ± 1.3 K/μL) (Table 3, Fig. 5d). Total white blood cell count was positively correlated with H:L ratio (*ρ* = 0.59) and HA:L ratio (*ρ* = 0.67) at the baseline sampling point (Fig. 3).

Log10-ratios of H:L and HA:L in cottonmouths both responded significantly to acute stress with an increase in values at the acute stress sample but decreased at the DEX sample, although this value was elevated relative to values at the baseline sample (Table 3, Fig. 5b and 5c). We found no overall population differences for log10-H:L and log10-HA:L ratios (Table 2). H:L and HA:L ratios were positively correlated with each other at baseline (*ρ* = 0.91), acute (*ρ* = 0.66) and DEX (*ρ* = 0.87) sampling points (Fig. 3).

Cottonmouths from Seahorse Key had a significantly higher number of immature red blood cells on average (- = 12.7 ± 5.8) than Snake Key individuals (- = 5.1 ± 2.0) (Table 3). There was no difference in number of immature red blood cells found across sampling points (Table 2). Immature red blood cells were weakly negatively correlated with NAb (*ρ* = −0.46) at the acute sampling point (Fig. 3).

A complete summary of white blood cell values can be found in Table 4 (conventional units) and results of linear mixed model analyses in Table S1. We found the presence of gametocytes of the intra-erythrocytic *Hemoproteus* sp. in blood films was higher for the Seahorse Key population (66.7%, *n* = 6) compared to Snake Key (7.7%, *n* = 13). We did not find snakes infected with haemoparasites to show a discernable pattern in their corticosterone response.

## Discussion

The sudden cessation of waterbird nesting on Seahorse Key and partial shift of nesting activities to Snake Key was a major disturbance for the inhabiting snake populations, which provided a unique opportunity to assess the effects of resource availability on the stress response and immune functions of cottonmouth snakes under natural conditions. While we were fortunate in our ability to take advantage of this ‘natural experiment’, we must clearly state that it was not possible to provide all the necessary controls associated with standard experimental design such as replication at the population level, and we caution readers to this fact prior to the discussion of our findings. Our results indicate that the sudden shift in bird-provided food resources from Seahorse and Snake Key in 2015 have manifested as several important differences between the islands in physiological and immune biomarkers of cottonmouth snakes. Our findings support our predictions that food-restricted snakes from Seahorse Key showed decreased body condition, stunted release of corticosterone and glucose in response to stress and decreased investment in total cellular and NAb-mediated immunity. We did not find support for predicted differences in baseline corticosterone, clearance of corticosterone following DEX, packed cell volume, H:L/HA:L ratio and erythrocyte sedimentation rate. It is apparent from these results that some physiological biomarkers of stress and general health were not responsive to food deprivation in our study and did not provide information for making conservation assessments based on the health of individuals or population. Because of the variation we found in the response of biomarkers of individual health to starvation, we recommend the use of multiple biomarkers in concert and consideration of potential confounding factors in interpreting results to provide additional context for results from any one metric.

Although we found lower body condition in resource-limited cottonmouths from Seahorse Key, baseline corticosterone was not discernibly different between the two populations. This contrasts with our prediction and with studies reporting elevation of baseline corticosterone following food restriction as the ‘conserved’ pattern (Dickens and Romero 2013). The effects of starvation are complex, and studies have shown that extended periods of fasting may increase or decrease corticosterone depending on the stage of starvation (McCue 2010; Romero and Wikelski 2010). Increased circulating glucocorticoids are thought to be the proximate mechanism of death during the final stage of starvation (Sapolsky *et al*. 2000; McCue 2010), which we did not find in the potentially starved cottonmouths from Seahorse Key.

**Table 3:** Summary table of mean ± SD (SD = standard deviation), minimum (Min) and maximum (Max) values and sample size (*n*) of corticosterone, glucose, natural antibody (NAb) agglutination activity, white blood cell (WBC) counts, immature red blood cell (RBC) values, log10-heterophil to lymphocyte (H:L) ratios, log10-heterophil plus azurophil to lymphocyte (HA:L) ratios and erythrocyte sedimentation rate (ESR) for Florida cottonmouth snakes (*Agkistrodon conanti*) from Seahorse and Snake Key

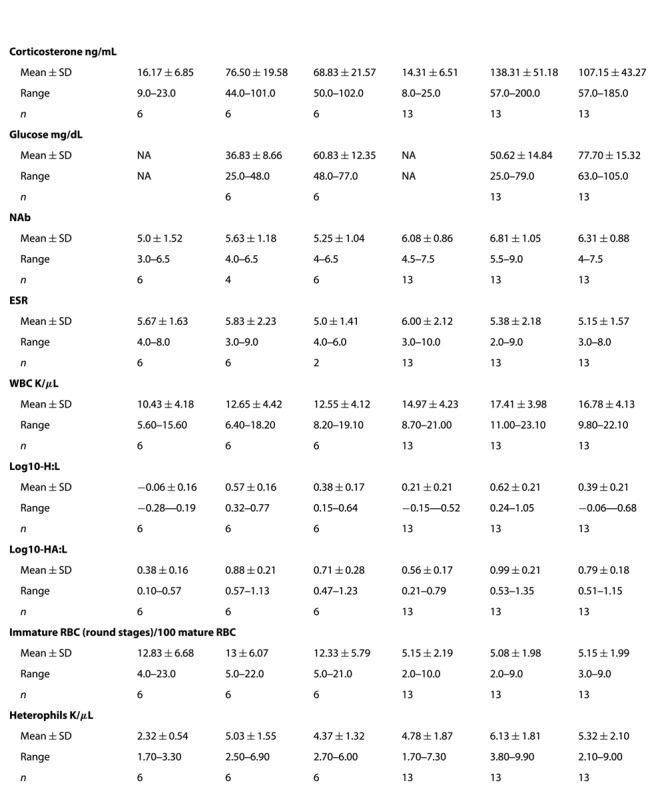
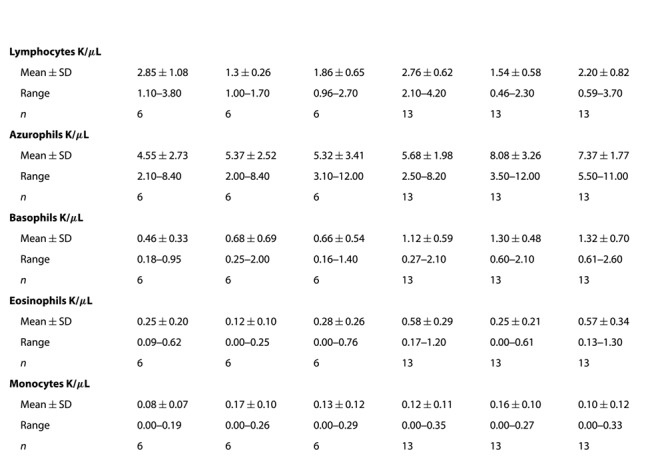

Seahorse Key has low food abundance and Snake Key has high food abundance. Each population was sampled at three sampling time points (baseline, acute and 60 min after the injection of dexamethasone (120 min post-capture, ‘DEX’ sample)

**Table 4:** Summary table of the mean (±SD) corticosterone values for each of five methods used for calculating the negative feedback success of cottonmouths on Seahorse Key (*n* = 6) and Snake Key (*n* = 13) following injection with dexamethasone and resulting Wilcoxon rank sum tests comparing the two snake populations

	Method 1	Method 2	Method 3	Method 4	Method 5
	Raw post DEX (ng/mL)	Difference from baseline (ng/mL)	Relative from baseline (%)	Reduction from acute (ng/mL)	Relative reduction from acute (%)
Seahorse Key $\tilde{\textrm{x}} $	68.79	52.82	451.02	7.8	8.48
Seahorse Key SD	21.59	27.24	375.35	17.63	22.55
Snake Key $\tilde{\textrm{x}} $	107.08	92.71	771.89	31.19	12.23
Snake Key SD	43.26	43.4	440.69	57.26	43.23
Wilcoxon W	14	15	21	35	35
Wilcoxon *P*	0.029^*^	0.036^*^	0.127	0.765	0.765

Seahorse Key has low food abundance and Snake Key has high food abundance. An asterisk denotes a significant *P* value with alpha set at 0.05

Low body condition in colubrid snakes is typically associated with higher circulating baseline corticosterone (Moore *et al*. 2000; Waye and Mason 2008; Lind *et al*. 2018). In large-bodied pit vipers, however, studies have found no relationship between corticosterone and body condition (Capehart *et al*. 2016; Lind *et al*. 2018). Colubrid snakes, which may need to feed more often, also show increased baseline corticosterone in populations with fewer food resources (Palacios *et al*. 2012) or after food restriction (Holden *et al*. 2019). This study adds to a growing body of evidence that baseline plasma corticosterone alone should not be used to assess chronic stress in pit vipers. Some species of rattlesnakes maintain similar baseline corticosterone concentration throughout the year, despite differential resource availability across seasons (Taylor *et al*. 2004; Lind *et al*. 2010). Bird-provided food for *A. conanti* is cyclical (Wharton 1969), but corticosterone values do not follow cycles of resource availability (Lind *et al*. 2018). When baseline corticosterone does change, increases are associated with transitions to reproductive stages (Lind *et al*. 2018). Chronic stress via food limitation may be part of the normal experience for pit vipers, resulting in resistance to increased baseline corticosterone due to long-term evolutionary adaptation to limited resource availability (Boonstra 2013). In the congeneric *A. piscivorus*, Graham *et al*. (2008) found higher plasma corticosterone in spring, but those plasma concentrations may not represent true baseline levels because collections were post-anaesthesia (Romero and Reed 2005). Additionally, attempts to induce increased baseline corticosterone *via* repeated stressors in wild rattlesnakes held in captivity failed (Claunch *et al*. 2017b). Because of the taxonomic consistency of disassociation between baseline corticosterone and chronic stress in pit vipers thus far, our observation may be more related to phylogeny than an effect of adaptation to resource shifts.

Interestingly, cottonmouths from Seahorse Key showed a dampened corticosterone response to acute stress relative to cottonmouths from Snake Key. This contrasts with literature on reptiles that reports an inverse relationship between acute corticosterone response and food availability (see Dunlap and Wingfield 1995; Kitaysky *et al*. 2001; Romero 2001; Romero and Wikelski 2001; Romero and Wikelski 2010; Neuman-Lee *et al*. 2015). It is also assumed that lower, not higher, plasma corticosterone concentrations during acute stress are associated with greater fitness (Breuner *et al*. 2008; Bonier *et al*. 2009). Blunted stress response was observed in the congeneric *A. contortrix* living closest to roads, a potential chronic stressor (Owen *et al*. 2014). Additionally, a recent meta-analysis on reptiles found acute corticosterone response to be positively associated with net primary productivity, a coarse measure of resource availability (Jessop *et al*. 2013). When exposed to a chronic stressor, a prolonged stress response becomes detrimental to an animal when the acute stress response becomes impaired or downstream effects reduce reproductive ability and/or decrease survival (Boonstra 2013). One method for vertebrates to endure chronic stress is to attenuate the stress response by downregulating corticosterone release when encountering acute stressors (Rich and Romero 2005). This strategy may prevent corticosterone itself from disrupting normal functions (Sapolsky *et al*. 2000; Wingfield and Romero 2001) and is partially supported by our findings. Our interpretation is limited by the fact that we do not know if a dampened corticosterone response is truly adaptive or phenotypic plasticity due to the changes in resource availability.

We observed slight decreases in corticosterone 1 h following DEX administration, as expected (Romero 2004; Sopinka *et al*. 2015). This was observed in both populations, which provides some evidence against dysregulation of HPA in chronically stressed snakes on Seahorse Key. Interestingly, using Methods 1 and 2 of calculating negative feedback function we found Snake Key cottonmouths to have higher corticosterone concentrations following injection with dexamethasone contrary to our predictions. However, calculation Methods 1 and 2 do not take into account changes in corticosterone concentrations during the acute stress response and for this reason are discouraged as metrics of negative feedback success (Lattin and Kelly 2020). The method currently recommended is Method 5 (Lattin and Kelly 2020), which incorporates acute stress data, and in our study did neither reveal a significant difference between populations nor did it correlate with Methods 1 and 2. It is important to note, however, that a 1-h time frame may be too short to saturate glucocorticoid receptors in cottonmouths and cause more extreme decreases in endogenous corticosterone production as seen in other studies (Romero and Wikelski 2010; Nevarez *et al*. 2011).

Glucose is the most commonly measured metabolite in the study of starvation, but blood glucose response to starvation in snakes varies among species (McCue *et al*. 2012). Cottonmouths on Seahorse Key had significantly lower glucose concentrations when responding to acute stress. Due to the lower limit of the glucometer, we were unable to assess differences in baseline glucose levels. Baseline glucose in all snakes from both locations were notably low suggesting snakes were fasted at the time of sampling (Wharton 1969) and/or that these concentrations reflect normal glucose homeostasis during the active season. Our results support the majority of previous investigations of glucose in reptiles that showed increased glucose values with acute stress (Gangloff *et al*. 2017; Neuman-Lee *et al.*, 2020; this study, but see Flower *et al*. 2015). We found corticosterone and glucose to be significantly positively correlated at only the acute sampling point. These findings support the suggestion of Neuman-Lee *et al.* (2020) that corticosterone has a complex but not direct role in mobilizing glucose stores when encountering an acute stressor. Also, we found no relationship between glucose levels and body condition. Sparkman *et al*. (2018) suggested lower glucose concentrations were influenced by limited prey availability in insular relative to mainland populations of *Coluber constrictor* and *Pituophis catenifer*. All viperids tested to date (*Crotalus atrox* and *Bitis gabonicus*) (McCue *et al*. 2012; Webb *et al*. 2017) experienced a decrease in blood glucose following food restriction, supporting our results.

The measured circulating immune capacity of cottonmouths on Seahorse Key was altered relative to those on Snake Key, as Seahorse Key snakes had fewer total white blood cells and less haemagglutination ability/NAb activity. Apart from differences of resource availability and effects from physiological stress, presence of parasites or pathogens on each island may be partially responsible for the observed differences in immune functions. Higher white blood cell counts for snakes on Snake Key, as reflected in concurrently higher azurophils, basophils, eosinophils and heterophils, could be attributed to active leukocyte responses to antigenic stimulation (Stacy *et al*. 2011). Total white blood cell counts for snakes on Seahorse Key, although low relative to Snake Key individuals, were higher than those reported for congeners (*A. piscivorus*) from North Carolina (Minter *et al*. 2013). The innate immune system is the first line of defence and may be the most ecologically relevant immune measure in ectotherms (Moeller *et al*. 2013) through early resistance against infection (Ochsenbein and Zinkernagel 2000; Matson *et al*. 2005). Increased prevalence of a common snake haemoparasite (*Hemoproteus* sp.) may be explained by lower NAb activity or leukocyte number in cottonmouths from Seahorse Key (67% infected; *n* = 6). However, presence of the haemoparasite is typically considered an incidental finding (Stacy *et al*. 2011), and NAb activity (Sparkman and Palacios 2009) and leukocyte number (Minter *et al*. 2013) were unrelated to parasite loads in garter snakes and Northern cottonmouths, respectively.

Regarding resource availability, there is precedent for differences in NAb activity in snakes. Luoma *et al*. (2016) found NAb activity increased with digestive activity in a colubrid snake, *Pantherophis guttatus*. And Madsen *et al*. (2007) suggested increased NAb activity may be an adaptive response to the pathogenic effects of food items in water pythons, *Liasis fuscus*. Theoretically, it is possible that the shift in diet of cottonmouths on Snake Key from feeding on live prey to regurgitated fish carrion or the continued antigenic stimulation of regular feeding might have led to our observed difference in NAb activity between islands, but this is speculation as we do not have data on the pathogenicity of food items. The body condition index of individual cottonmouths positively correlated with NAb activity and lends further support to the general link between energy availability and investment in immunity (but see Moeller *et al*. 2013; Davies *et al*. 2016). Interestingly, there was both a decreased investment in NAbs and a decoupling of NAb response to acute stress in Snake Key snakes, as those on Snake Key had relatively higher NAB activity at all measurements and increased NAb activity in response to acute stress. This partially corroborates laboratory findings from exogenous corticosterone application on lizards, where chronic application (which may mimic food deprivation stress in lizards) led to decreased NAb activity, but low-dose acute application (which may mimic acute capture stress) led to increased function (McCormick *et al*. 2015), and in food-deprived colubrid snakes with lower NAb activity (Holden *et al*. 2019). It is important to emphasize, however, that in both aforementioned studies baseline corticosterone was increased in the chronic or food-deprived group(s), respectively, while the food-deprived cottonmouths on Seahorse Key did not show changes in baseline corticosterone. While the overall immune responses were altered in chronically stressed cottonmouths on Seahorse Key, leukocyte responses, at least in terms of changes in abundance, were presumptively still functional with acute stress.

The resource-limited cottonmouths on Seahorse Key showed higher numbers of immature red blood cells across all time points, providing evidence that these snakes show active erythroid regeneration despite the absence of anaemia or differences in packed cell volume in both snake populations. It is possible that cottonmouths on Seahorse Key may have exhibited masked anaemia from various potential underlying causes due to dehydration; however, both populations should experience dehydration at similar rates from reduced freshwater availability due to a reliance on rainfall for drinking (Sandfoss *et al.*, 2019). Since this information is unknown in our study, we consider this a confounding factor which limits our ability to draw substantiated conclusions. Ongoing erythroid regeneration is a non-specific indicator of active stimulation of red blood cell production in haematopoietic tissues in response to numerous potential underlying causes for loss, lysis or reduced production of red blood cells and is thus considered a general positive diagnostic finding in reptiles (Stacy *et al*. 2011). Absent erythroid regeneration has been associated with advanced or end-stage starvation and poor prognosis in chronically debilitated loggerhead sea turtles (*Caretta caretta*) with low packed cell volume/anaemia (Stacy *et al*. 2018).

Ratios of H:L (and HA:L) and erythrocyte sedimentation were not distinguishable with respect to chronic stress in cottonmouths. H:L ratio has been suggested as a metric in assessing chronically stressed animals because they may remain elevated during stress before the HPA axis is exhausted (Davis and Maney 2018). However, this suggestion may rely on the elevation of baseline corticosterone for some duration under chronic stress in the species tested. Under this assumption, it is possible that a lack of population difference in H:L—despite a positive association with corticosterone—is due to lack of an observed population difference in baseline corticosterone or effective response in chronically stressed Seahorse Key snakes. In two closely related species of colubrid snake, no correlation was found between H:L and corticosterone in *Thamnophis elegans* (Sparkman *et al*. 2014), but was found in *T. sirtalis*; however, this relationship varied with time post-capture (Gangloff *et al*. 2017). We found no correlation between corticosterone and H:L at all sampling time points, which supports previous work on the flexibility and complexity of snake physiology in response to environmental conditions (Sparkman *et al*. 2014; Gangloff *et al*. 2017). H:L is suggested to follow a lag time in response of leukocytes following stress (Davis and Maney 2018), but we saw increases in H:L within 1 h in both populations. Given that there were no differences in erythrocyte sedimentation rate in this study, we doubt confounding effects of possible underlying inflammation on our H:L ratios. The ratios of H:L and HA:L after acute stress showed increases that were overall similar to data reported in other reptile species (Kreger and Mench 1993; Sparkman *et al*. 2014; Gangloff *et al*. 2017). Differences in comparing to other studies include possible effects of restraint methodology for at least 1 h, species-specific variation, or a combination with other factors such as time of day or season, and methodological differences (e.g. sample handling, sample processing or haematological analyses). Inclusion of azurophils in our comparisons of white blood cell ratios (HA:L) did not provide meaningful differences from ratios without azurophils (H:L). Azurophils—unique to squamates—are thought to be homologous to mammalian neutrophils (Montali 1988), but their functions and responses in disease or stress remain poorly understood.

Snakes on Seahorse Key were found to be in low body condition and exhibited a dampened corticosterone, glucose, white blood cell count and NAb activity following acute capture stress relative to Snake Key. This may demonstrate an energy-saving phenotype for chronic stress following long-term food restriction in cottonmouths. As cottonmouths have low-energy life histories (McCue and Lillywhite 2002) and are typically infrequent feeders, they are likely adapted for long periods without food. It could benefit a food-restricted cottonmouth to dampen corticosterone responses to acute stress to prevent additional energy mobilization via gluconeogenesis when energy stores are already low. Additional energy might be conserved by decreasing overall immune cell production, although we did not see a decoupling of the immune cell mobilization response to acute stress. The increases in leukocyte numbers in response to acute stress lend evidence for the importance of acute stress or corticosterone having a preparative or stimulating role for the immune system (Sapolsky *et al*. 2000). The context-dependent nature of stress on the immune system is important to consider (Dhabhar 2009), as chronic stress typically has immuno-suppressive effects. Because of our study design, we cannot distinguish whether acute stress or the increase in corticosterone was responsible for inducing changes.

### Implications for conservation

It is important to understand which metrics might be important in determining chronic stress across species with varying life histories. There is no general consensus on a gold standard in the investigation of responses to stress across species (Dickens and Romero 2013), making reliable endpoints difficult to find for use in monitoring conservation efforts. We believe our study provides a unique and valuable assessment of the physiological response of animals to natural ecological disturbance—largely due to the sudden and significant shift in food resources between two populations of snakes within a well-studied system (Cooke and O’Connor 2010). Long-term population monitoring is expensive, logistically challenging, and in many cases, may not be initiated until population declines are observed after a perturbance. Blood-derived metrics can be collected opportunistically and used to supplement routine monitoring of a population or as justification for focused management of certain populations/areas (Cooke and O’Connor 2010). We have shown that although multiple biomarkers are important to collect and consider in context of a given *in situ* population, meaningful results can be attained from blood samples of relatively few individuals. We have also demonstrated that popular biomarkers (e.g. baseline corticosterone or H:L ratios) are not ubiquitous indicators of chronic stress in wild animals.

Aside from expanding knowledge of chronic stress effects in an understudied group of ectothermic vertebrates, our study also provides evidence for metrics of chronic stress in pit vipers: body condition index, acute stress response, white blood cell counts and NAb. These metrics may be suitable for monitoring species of pit vipers that are likely to be affected by climate change (e.g. montane rattlesnakes) or various human disturbances (e.g. timber rattlesnakes *Crotalus horridus* and Eastern Massasauga rattlesnakes *Sistrurus catenatus*). We advocate for the continued and concurrent study of different metrics and endpoints in chronically stressed animal populations *in situ*, especially of understudied taxonomic groups, and for the continuous development and validation of biomarkers for facilitating future investigations of health status and the effects of ecological changes in animals with similar life history traits.

## Funding

This work was supported by the University of Florida’s May interdisciplinary grant awarded to MR Sandfoss and N Claunch. Additional funding was provided by the University of Florida’s Seahorse Key Fellowship awarded to MR Sandfoss.

## Supplementary Material

Sandfoss_et_al_2019_Cons_Physiol_Second_Revision_coaa031
