# Supplementary material for: A tale of two islands: evidence for impaired stress response and altered immune functions in an insular pit viper following ecological disturbance
Source: Conserv Physiol. 2020 May 3;8(1):coaa031. doi: 10.1093/conphys/coaa031 (PMC7196672; doi:10.1093/conphys/coaa031)
Supplement: Sandfoss_et_al_2019_Cons_Physiol_Second_Revision_coaa031 [file sandfoss_et_al_2019_cons_physiol_second_revision_coaa031.docx]

**Supplemental Tables**

Table S1. Results of repeated measures ANOVA testing for effects of sampling time point (Sample), population, and their interaction for measures of leukocyte cell types from whole blood samples collected from Florida cottonmouth snakes (*Agkistrodon conanti*) from Seahorse (*n* = 6) and Snake Keys (*n* = 13). Each coefficient is calculated in relation to Seahorse Key Population, Baseline Sample, or Snake Key * Baseline Sample. Population refers to differences between population (Seahorse and Snake Keys). Seahorse Key has low food abundance and Snake Key has high food abundance. Sample refers to the three sampling time points (baseline, acute, and 60 minutes after the injection of dexamethasone (120-minutes post-capture, “DEX” sample). A superscript “S” indicates statistically significant at the 0.05 level.

| **Blood Value** | **Variable** | **F** | **df** | ***P*** | ***Coef*** | ***SE*** |
| --- | --- | --- | --- | --- | --- | --- |
| Basophils | Population | 10.307 | 1,34 | 0.005^S^ |  |  |
|  | *Snake Key* |  |  |  | *0.663* | *0.286* |
|  | Stress Sample | 3.099 | 2,34 | 0.058 | - | - |
|  | Population * Stress Sample | 0.01 | 2,34 | 0.99 | - | - |
| Monocytes | Population | 0.002 | 1,17 | 0.964 | - | - |
|  | Stress Sample | 1.682 | 2,34 | 0.201 | - | - |
|  | Population * Stress Sample | 0.416 | 2,34 | 0.663 | - | - |
| Eosinophils | Population | 140.706 | 1,17 | 0.005^S^ |  |  |
|  | *Snake Key* |  |  |  | *0.333* | *0.129* |
|  | Stress Sample | 7.992 | 2,34 | 0.001^S^ |  |  |
|  | *Acute* |  |  |  | *–0.125* | *0.146* |
|  | *DEX* |  |  |  | *0.028* | *0.146* |
|  | Population * Stress Sample | 0.703 | 2,34 | 0.502 | - | - |
| Azurophils | Population | 4.058 | 1,17 | 0.06 | - | - |
|  | Stress Sample | 8.767 | 2,34 | 0.001^S^ |  |  |
|  | *Acute* |  |  |  | *0.817* | *0.891* |
|  | *DEX* |  |  |  | *0.767* | *0.891* |
|  | Population * Stress Sample | 0.43 | 2,34 | 0.654 | - | - |
| Heterophils | Population | 4.843 | 1,17 | 0.042^S^ |  |  |
|  | *Snake Key* |  |  |  | *2.460* | *0.864* |
|  | Stress Sample | 9.413 | 2,34 | 0.001^S^ |  |  |
|  | *Acute* |  |  |  | *2.717* | *0.734* |
|  | *DEX* |  |  |  | *2.050* | *0.734* |
|  | Population * Stress Sample | 1.762 | 2,34 | 0.187 | - | - |
| Lymphocytes | Population | 0.488 | 1,17 | 0.494 | - | - |
|  | Stress Sample | 16.822 | 2,34 | <0.0001^S^ |  |  |
|  | *Acute* |  |  |  | *–1.550* | *0.393* |
|  | *DEX* |  |  |  | *–0.990* | *0.393* |
|  | Population * Stress Sample | 0.11 | 2,34 | 0.896 | - | - |
